# Revolutionizing heart failure management: remote speech analysis as a noninvasive biomarker—letter to the editor

**DOI:** 10.1097/MS9.0000000000000863

**Published:** 2023-06-05

**Authors:** Maria Waseem, Fatima Haq, Muneeb Ullah Jan, Usha Kumari, Aarash Khan

**Affiliations:** a Dow University of Health Sciences, Karachi; b Department of Cardiology, Lady Reading Hospital, Peshawar, Pakistan; c Herat University, Herat, Afghanistan

Heart failure (HF) is a syndrome that is becoming more common and is the main reason for both first hospitalizations and re-admissions^1^. Around 26 million individuals globally suffer with HF, and this number is predicted to increase even more^2^. Surprisingly, HF is the leading cause of readmission in the adult population, accounting for up to 25% of patients being readmitted within 30–60 days. It could be categorized as a “malignant condition” based on the poor prognosis^1^. Patients with HF may be stable, but acute decompensated HF, which causes the majority of HF hospitalizations, can develop as the underlying diseases progress or due to other health changes^2^. Acute decompensation is frequently accompanied by congestive symptoms^1^. Despite modern treatments, hospital admission rates for HF are still high. Each HF-related hospitalization raises the risk for subsequent hospitalization occurrences. Therefore, to deal with the growing burden of HF, advances in outpatient care are required. Voice evaluation has recently been proposed as a low-cost, noninvasive tool for coronary artery disease. Patients with HF or pulmonary congestion that has decompensated have been shown to have changes in their vocal patterns. Additionally, among HF patients, alterations in voice features were linked to unfavourable outcomes. These results point to the possibility of voice as a novel biomarker for noninvasively monitoring HF patients^2^.

Amir *et al*.^3^ assessed the efficacy of automated speech analysis technology in detecting pulmonary fluid in the patients of acute decompensated HF (ADHF) and found that it can recognize voice alterations reflective of the degree of HF, putting forward a great possibility of effectual in-person evaluation and remote follow up of ADHF patients. The approach brings forth the possibility of an equally effective noninvasive vocal biomarker for evaluation of clinical conditions which tend to alter the functioning of vocal folds. During phonation, the air is forced through the vocal folds as they are approximated by muscles attached to the arytenoids. The combined effect of the approximated vocal folds and the forceful flow of air cause the folds to vibrate and produce sound. The HF-sensitive oedema of vocal folds may interfere with the usual phonation yielding appreciable acoustics. The deviated function of oedematous vocal folds may also result in increased pauses in speech corresponding to the compensation of inappropriate subsequent phonation. Speech and pause alterations together with acoustic speech analysis have provided promising evidence of better disease progression monitoring and diagnosis in patients of ADHF.

Schöbi *et al*.^2^ evaluated speech and pause alterations in voice recordings of acute and stable patients and found out that pause ratio was 14.9% increase in patients of acute HF while it also positively correlated with chemical biomarkers.

ADHF clinically presents initially with varying degree of dyspnoea, orthopnea or systemic oedema corresponding to a number of possible causes, which are also used for risk stratification and devising management plans predicting the long-term mortality^4^. High in-hospital mortality, frequent re-admissions and subsequent poor clinical course of disease is associated with the patients of decompensated HF. Hence a thorough evaluation of initial presentation, timely diagnosis and optimal management must be taken into account for better prognosis, the facilities of which are usually not available in rural areas of Pakistan. Assessments usually employed in evaluating ADHF include a Full Blood Count, basic metabolic profile, liver function tests, troponin, brain natriuretic peptide, a chest radiograph and an electrocardiogram; however, they do not appear to significantly reduce readmission rates^5^. Martindale *et a*l.^6^ have found that bed side lung ultrasound and echocardiography may be the most useful tests for timely diagnosis but signs of decompensation are still often missed despite of frequent monitoring with adequate facilities available.

Introduction of remote automated speech analysis technology in diagnosing and managing ADHF in rural areas of Pakistan may be revolutionary corresponding to more than half of the disease burden arising from distant rural areas together with the lack of medical expertise and appropriate facilities to carry out the usual assessments in the vicinity of these areas. Together with this, the inoculation of the technology for remote follow-ups in busy urban set-up may decrease the risk of re-hospitalizations.

Several hinderances lie in the path of implementing the method of remote speech analysis in-hospital settings of Pakistan. There is an absence of technologically trained staff and healthcare professionals who can effectively utilize this novel technology through smartphones applications, computer software and servers. Being an underdeveloped and low-income country Pakistan lacks the monetary resources required for executing this useful noninvasive method of monitoring patients suffering from diseases such as decompensated HF etc. The availability of relevant hardware equipment, gadgets and software is limited because the technique of remote speech analysis is quite recent and not well known. Ethical challenges regarding the misuse of patients’ speech data also exist.

The potential benefits of employing the technologies used in remote speech analysis include noninvasiveness, catering patients residing in far fletched rural areas, time effectiveness and accuracy. It is therefore important that suitable measures are taken for implementing this method in Pakistan. Skilled individuals can be hired in hospitals so that they handle the technological system. Government can work together with private sector hospitals and allocate funds to meet the expense of the application and equipment. Assistance from healthcare workers in other countries running this novel system can be sought.

## Ethical approval

Ethical approval was not required for this commentary article.

## Consent statement

Informed consent was not required for this commentary article.

## Sources of funding

None to declare.

## Author contribution

M.W.: idea conceptualization, writing original draft, study design. F.H.: writing original draft, project administration. M.U.J., writing original draft, proof reading, formatting. U.K.: project administration, proof reading, formatting, reviewing. A.K.: editing, project administration, proof reading.

## Conflicts of interest disclosure

No conflict of interest to disclose.

## Research registration unique identifying number (UIN)

Not applicable.

## Guarantor

Usha Kumari.

## Data availability statement

Not applicable.

## Provenance and peer review

Not commissioned, externally peer-reviewed.
